# Brief exposure to Swedish snus causes divergent vascular responses in healthy male and female volunteers

**DOI:** 10.1371/journal.pone.0195493

**Published:** 2018-04-18

**Authors:** Lukasz Antoniewicz, Mirza Novo, Jenny Bosson, Magnus Lundbäck

**Affiliations:** 1 Karolinska Institutet, Department of Clinical Sciences, Division of Internal Medicine, Danderyd Hospital, Stockholm, Sweden; 2 Umeå University, Department of Public Health and Clinical Medicine, Division of Medicine/Respiratory Medicine, Umeå, Sweden; 3 Karolinska Institutet, Department of Clinical Sciences, Division of Cardiovascular Medicine, Danderyd Hospital, Stockholm, Sweden; National Yang-Ming University, TAIWAN

## Abstract

**Introduction:**

The use of Swedish oral moist snuff, known as snus, has for a long time been limited to the Scandinavian countries. With declining cigarette sales in the western world, tobacco companies have looked to the development of alternative tobacco products. In 2006 snus products were launched in the US. Even though several studies have demonstrated negative health effects, snus is often depicted as harmless.

The aim of the present study was to investigate acute vascular effects of snus as measured by arterial stiffness as well as blood pressure and heart rate.

**Methods:**

Two separate randomized double-blind crossover studies with the same study design were pooled for analysis. Twenty-nine healthy snus-users (17 females, 12 males) were included. Snus (Göteborgs Rapé) and tobacco free snus (Onico) were administered in a randomized order at two separate visits. Arterial stiffness, blood pressure and heart rate were measured at baseline as well as every five minutes for 40 minutes during exposure. Following snus removal, measurements continued for 30 minutes post exposure. Arterial stiffness was measured using pulse wave velocity (Vicorder) and pulse wave analysis (Sphygmocor).

**Results:**

Compared to placebo, snus significantly increased systolic and diastolic blood pressure as well as heart rate, however, only in females (p = 0.004, p = 0.006 and p<0.001 respectively). No changes were seen in arterial stiffness measurements in either gender.

**Conclusion:**

We observed an increase in blood pressure and heart rate only in females, but not in males due to snus usage as compared to placebo. This novel finding was surprising and needs to be further investigated considering most of the earlier studies have mainly focused on male snus users and the increasing usage of snus among females.

## Introduction

Cigarette smoking is one of the leading causes of premature death and is associated with increased mortality and morbidity due to cardiovascular disease [1]. The WHO estimates that 6 million people die annually due to cigarette smoke exposure [2]. The firmly established association between cigarette smoking and impaired health in combination with increased public awareness has caused cigarette sales to drop dramatically in western nations. Therefore, large transnational tobacco companies have started to look to alternative tobacco products such as Swedish moist snuff, also known as snus [3]. Since 2007 Marlboro, Skoal and Camel have all launched Swedish style snus in the US, which has been aggressively marketed as a potentially healthier alternative to cigarette smoking with advertisement primarily targeting smokers in situations where smoking is prohibited [4].

Historically, much of the attention from the research community has been focused on the potential adverse effects of snus on the oral mucosa as it is placed between the lip and gums. However, no strong associations between snus usage and oral cancer have been found [5]. Yet, in recent years compelling new evidence of the possible cardiovascular effects of snus has been established. Snus use has been linked to heart failure, development of type 2 diabetes and increased mortality following myocardial infarction and stroke [6–9]. Furthermore, after myocardial infarction, discontinuation of snus use was associated with an almost fifty percent decreased risk in mortality [9]. These findings are not without controversy and several studies present contradicting results regarding the overall risk for cardiovascular disease [10–13].

Swedish Match, the largest snus manufacturing tobacco company in Scandinavia and the second largest in the US, has recently submitted an application for snus to be sold as a modified risk tobacco product (MRTP) to the U.S. Food and Drug Administration (FDA) [14]. This would allow the company to market their products as a healthier alternative to smoking as well as allow for removal of certain warning labels. Several studies, sponsored by Swedish Match, dismiss the risk and severity of snus use and try to shift the focus of discussion to snus as a smoking cessation aid [15–19]. However, no clinical studies to date have demonstrated that snus successfully facilitates smoking cessation [15, 20, 21].

Furthermore, snus has been linked to increased blood pressure, increased heart rate and endothelial dysfunction, the latter demonstrated by ultrasound assessment of the brachial artery [22–24]. To our knowledge, no placebo-controlled exposure study investigating the acute effects of Swedish snus on arterial stiffness has been performed to date.

## Materials and methods

### Study design and subjects

**Fig 1:**
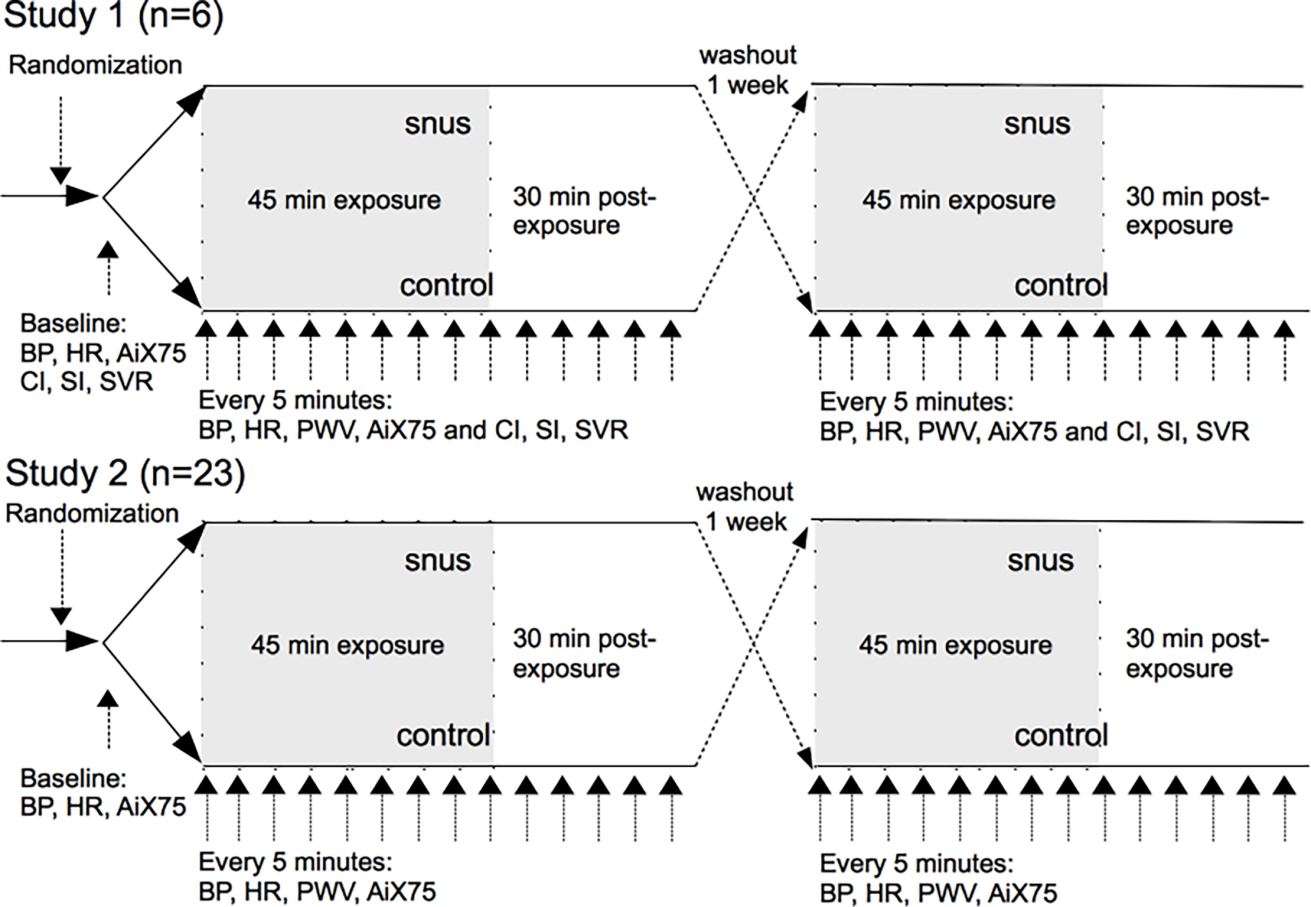
Study design for study 1 and 2. Double blinded crossover study. Blood pressure (BP), heart rate (HR) as well as pulse wave velocity (PWV) and arterial index (AiX75) were measured every 5 minutes in both studies. Additionally, thoracic electrical bioimpedance measurements with stroke volume index (SI), cardiac index (CI) and systemic vascular resistance (SVR) were performed in study 1.

In two randomized, double blind, crossover studies, healthy young volunteers, who use snus on a daily basis, attended on two occasions at least one week apart (Fig 1). On the two study days they were exposed to conventional snus (Göteborgs Rapé™, nicotine content: 8mg/g) and a flavored, plant fiber-based snus pouch free of tobacco and nicotine (ONICO™), which is often used as a snus cessation aid. The volunteers abstained from alcohol for 24 hours and from food, tobacco and caffeine containing drinks for at least 12 hours before both exposures. Following 20 minutes of rest a pouch of snus or non-tobacco/nicotine control was placed under the upper lip of the volunteers. Systolic and diastolic blood pressure (SBP, DBP respectively), heart rate (HR) as well as arterial stiffness, assessed using pulse wave analysis (PWA) and pulse wave velocity (PWV), were measured every five minutes for 45 minutes and during 30 minutes after exposure.

Two separate studies were performed with the same study design (Fig 1). Subjects in both studies were recruited in 2010 and 2012 through leaflets on University campus Umeå, advertising in local media and through information on social media. One subject in study 1 participated in only one exposure and was therefore excluded from the study. In the first study six females (mean age ± SD: 27.3 ± 8.2 years, range: 22 years) were included and thoracic electrical bioimpedance (TEB) was also measured at the same time points. In the second study 23 healthy volunteers (males: n = 12, females n = 11, mean age 30.5 ± 5.5 years, range: 19 years) were included.

The exact same study-design was used in both studies and therefore pooled analysis for HR, BP, PWA and PWV could be performed for study 1 and 2. Because of technical reasons thoracic electrical bioimpedance measurements were only obtained in study 1 and were therefore analyzed separately.

The study protocols were approved by the local Human Research Ethics Committee at Umeå University, Umeå, Sweden. All subjects have accepted and signed a written informed consent in accordance with the Declaration of Helsinki.

### Arterial stiffness

Measurement of arterial stiffness was performed in a quiet, temperature controlled room by a blinded operator with all volunteers resting in a semi-supine position. Following 20 minutes of rest, systolic and diastolic blood pressures were measured in duplicate using a semi-automated non-invasive oscillometric sphygmomanometer (Boso-Medicus, Boso, Jungingen, Germany) at baseline and every five minutes during 40 minutes of exposure and 30 minutes post exposure.

In accordance with manufacturer’s instructions, PWA was assessed with micro manometer applanation tonometry (Millar Instruments, Texas, USA) of the right radial artery at the wrist using the SphygmoCor™ system (AtCor Medical, Sydney, Australia). In brief, via a validated mathematical transfer function, pulse wave analysis obtains an aortic pulse pressure waveform from the radial artery. The arterial pressure waveform constitutes of a forward pressure wave (originated from the ventricular contraction) and a reflected wave caused by the peripheral vascular resistance. From this waveform, augmentation pressure (AP, expressed in mmHg) and augmentation index (AiX), both assessments of central arterial stiffness, are calculated. As AiX is inversely proportional to pulse rate, AiX is normalized for a HR at 75bpm (AiX75). Two independent waveform analyses were obtained from each volunteer. Measurements were accepted according to the SphygmoCor™ quality control criteria.

PWV, considered the “gold-standard” measurement of central arterial stiffness, was assessed using a Vicorder™ system (Skidmore Medical, Bristol, UK). This is a well-validated, non-invasive method of measuring PWV with small inflatable cuffs that are attached around the neck and upper thigh evaluating pulse waves in the carotid and femoral arteries simultaneously. The pulse transit time is assessed and is used along with the distance between the sites to calculate the pulse wave velocity.

### Thoracic electrical bioimpedance

Hemodynamic measurements were obtained by placing electrodes on the neck and chest of the volunteers using thoracic electrical bioimpedance (Hotman System, Hemo Sapiens Inc., MN, US). This non-invasive equipment was used to determine systemic vascular resistance index (SVRI), cardiac index (CI) and stroke index (SI).

### Statistical analysis

Statistical calculations were performed using SPSS Statistics (22.0, IBM Corporation, NY, US) and GraphPad Prism (7.0, GraphPad Software Inc., CA, US) software. Data was checked for skewness. Two-way ANOVA for repeated measures was performed for all dependent variables. If Mauchly’s test for sphericity was violated, Greenhouse-Geisser or Huynh-Feldt corrected results were presented depending on the highest epsilon value. For comparison of separate time points, paired samples T-test was applied. Independent samples T-test was used to analyze baseline characteristics. P-values of <0.05 were considered to be statistically significant. Blinded investigators performed all analysis.

## Results

Pooled analysis of study 1 and 2 was performed for heart rate (HR), systolic and diastolic blood pressure (SBP, DBP), pulse wave analysis (PWA), as well as pulse wave velocity (PWV). A separate analysis for cardiac index (CI), stroke index (SI) and systemic vascular resistance index (SVRI) was performed for study 1. There was no significant difference in baseline characteristics for females between those two studies (S1 Table).

Six women completed study 1 and 23 subjects (12 men and 11 women) completed study 2. The pooled analysis was performed on the two studies combined (n = 29, 12 men and 17 women, mean age: 29.9 ± 6.2 years). Study participants used snus for an average of 13.1 years ± 6.1 years. Weekly consumption was 5.1 ± 1.8 cans of snus. The portion form of snus was used by 89.7% and 10.3% used the loose type.

### Pooled analysis

Men had significantly higher SBP and PWV and lower AiX75 than women at baseline (Table 1). Women and men used approximately the same amount of snus per week and there was no significant difference in mean age or years of snus usage.

**Table 1:** Baseline values for men and women.

	Female (n = 17)	Male (n = 12)	p-values
Age [years]	28.6 ± 6.5	31.6 ± 5.3	0.211
Snus use [years]	11.9 ± 5.7	14.2 ± 6.5	0.386
Snus [cans/week]	5.1 ± 2.0	5.2 ± 1.5	0.909
SBP [mmHg]	108.3 ± 8.1	119.6 ± 9.1	0.003
DBP [mmHg]	66.9 ± 6,8	70.5 ± 7.9	0.282
HR [bpm]	57.0 ± 10.1	54.0 ± 11.6	0.108
AIx75 [%]	0.2 ± 10.1	-11.0 ± 6.1	0.001
PWV [m/s]	5.5 ± 0.8	6.3 ± 0.5	0.014

SBP = systolic blood pressure, DBP = diastolic blood pressure, HR = heart rate, AiX75 = arterial index for a heart rate at 75bpm, PWV = pulse wave velocity. Expressed as mean values ± SD.

**Fig 2:**
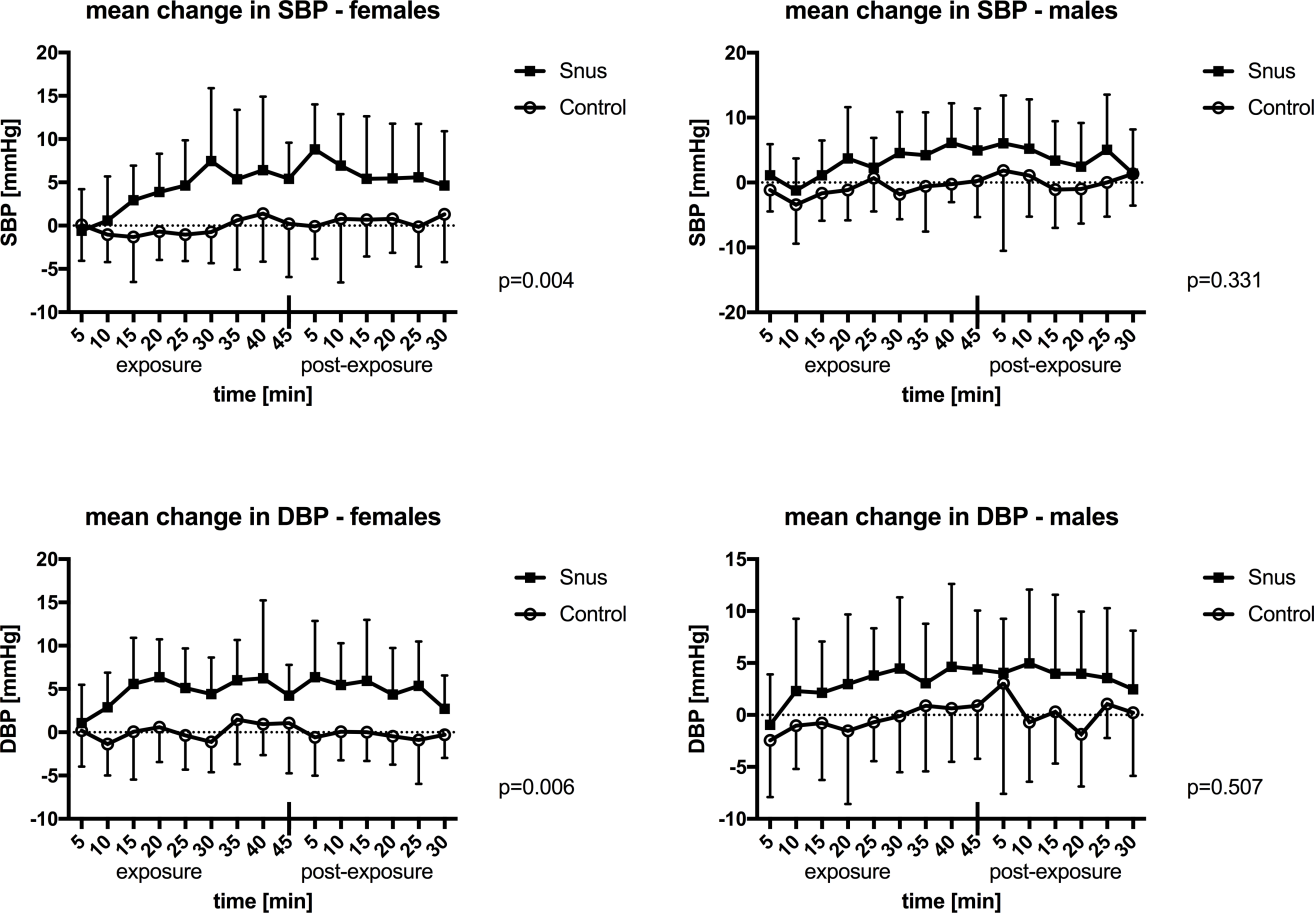
Mean change in systolic and diastolic blood pressure (SBP, DBP). Mean values with standard deviations during 45 minutes of exposure and 30 minutes post exposure to snus or control in males and females. P-values are presented for the interaction of time and exposure in multiple measures ANOVA.

There was no difference in baseline values prior to exposure to snus and placebo-control (S2 Table). SBP and DBP increased significantly five minutes following introduction of the snus pouch, when all subjects were analyzed with multiple measures ANOVA (p<0.001, p = 0.003 resp., S1 Fig). However, when analyzing women and men separately this increase in SBP and DBP was only significant for women (Fig 2). Mean changes in SBP and DBP separated for men and women with results from paired samples T-test are presented in S3 Table. For women, the mean increase in SBP/DBP during the last 25 minutes of exposure was 7.6 mmHg and 5.7 mmHg and for men 5.9 mmHg and 4.7 mmHg, respectively.

**Fig 3:**
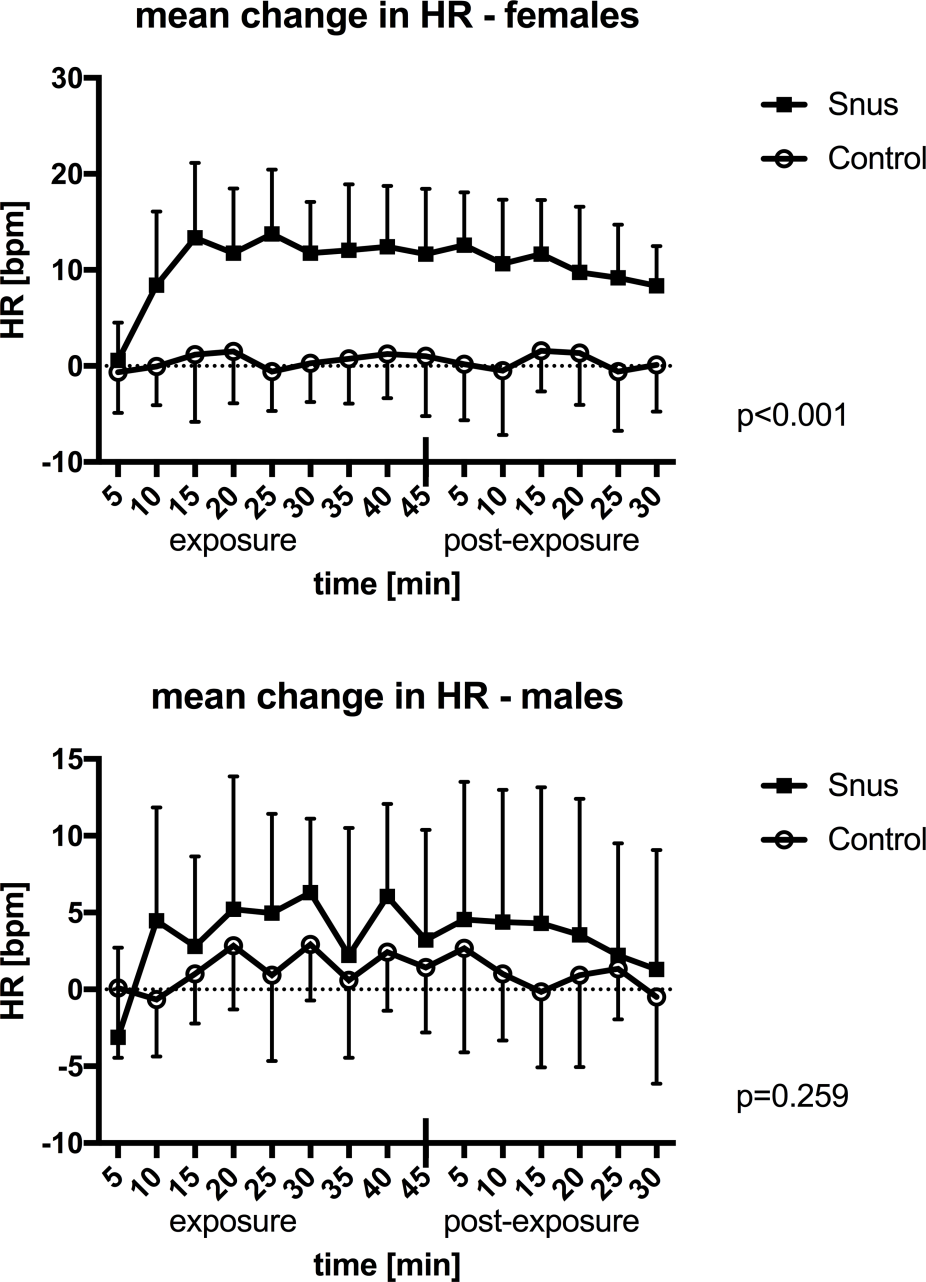
Mean change in heart rate (HR). Mean values with standard deviations during 45 minutes of exposure and 30 minutes post exposure to snus or control in males and females. P-values are presented for the interaction of time and exposure in multiple measures ANOVA.

HR increased significantly within 5 minutes following introduction of snus in females, and it remained significantly elevated during the whole post exposure period of 30 minutes (Fig 3). No significant change was seen in males when analyzed with multiple measures ANOVA (Fig 3). Mean changes in HR and paired samples T-test are shown in S3 Table. Mean increase in HR in women during the last 25 minutes of exposure was 11.9 bpm and for men 4.2 bpm.

**Fig 4:**
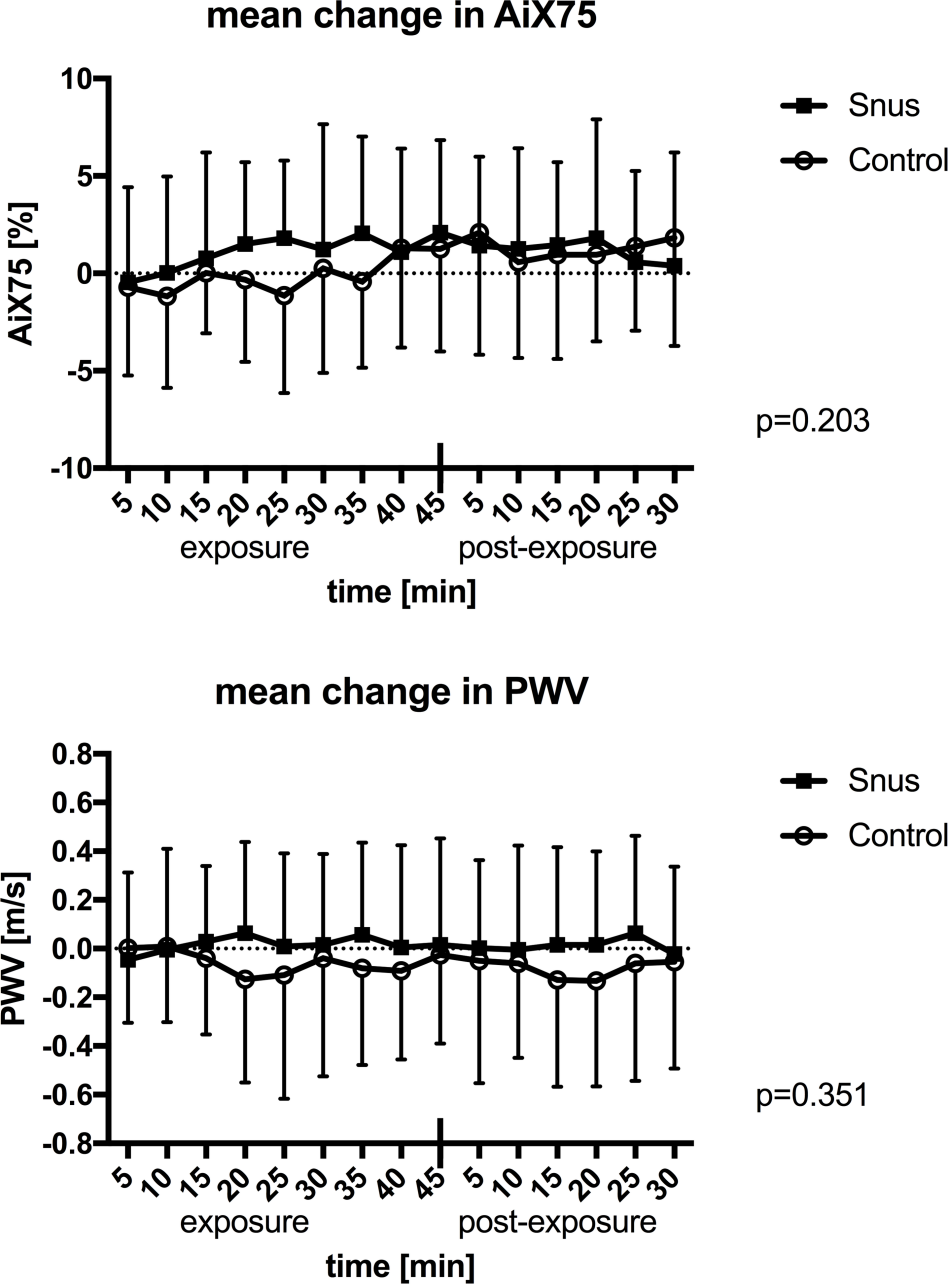
Mean change in arterial stiffness. Mean values for arterial index adjusted for a heart rate at 75 bpm (AiX75) and pulse wave velocity (PWV) with standard deviations during 45 minutes of exposure and 30 minutes post exposure to snus or control. P-values are presented for the interaction of time and exposure in multiple measures ANOVA.

AiX75 and PWV did not significantly change due to snus exposure as compared to placebo (Fig 4). There was no difference when analyzing separately for women and men (S2 Fig). However, when performing single time point analysis with paired samples T-test, women did show a significant increase during the last 25 minutes of exposure for snus (S3 Table). For men we observed a significant increase in AiX75 at the end of exposure and during the post-exposure period for placebo (S3 Table).

### Thoracic electrical bioimpedance results for study 1

**Fig 5:**
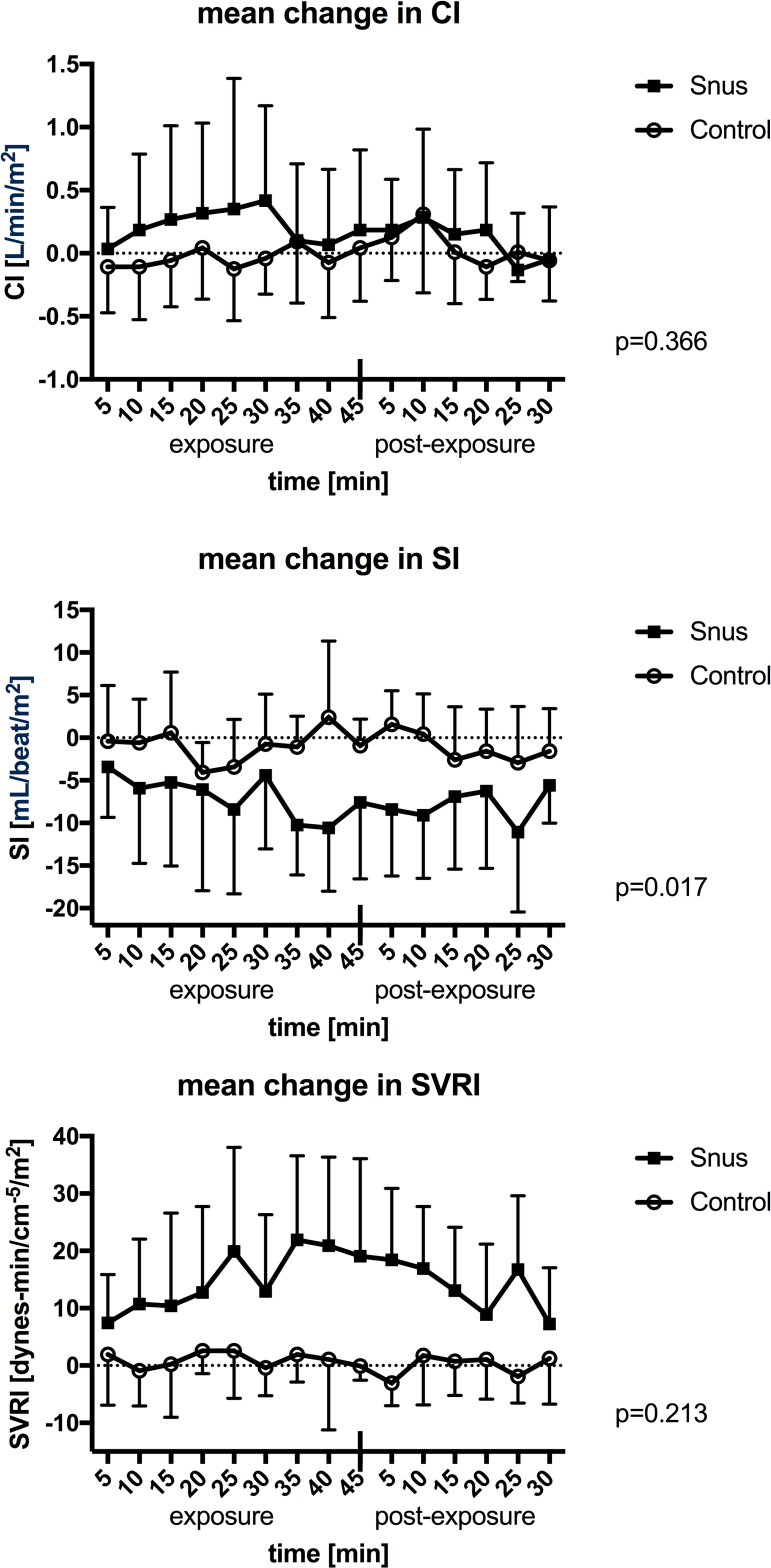
Mean change in cardiac index (CI), stroke volume index (SI) and systemic vascular resistance index (SVRI). Mean values with standard deviations during 45 minutes of exposure and 30 minutes post exposure to snus or control. P-values are presented for the interaction of time and exposure in multiple measures ANOVA.

Stroke volume index (SI) significantly decreased 35 minutes following introduction of snus and remained significantly altered 10 minutes into the post exposure period (Fig 5). SVRI increased 25 minutes after administrating snus, but this was not significant in multiple measures ANOVA. When analyzing with paired samples T-test, this effect was significant at time points 25, 35, 40 and 45min during exposure and during the first 15 minutes into the post exposure period (p-values ranging from 0.012 to 0.044). All other time points showed no statistical significance. Cardiac index (CI) did not significantly change over time in either exposure.

## Discussion

Short exposure to one pouch of snus caused an acute increase in systolic and diastolic blood pressure as well as heart rate in female and male volunteers, however to a lesser extent in males. Arterial stiffness measurements did not change following exposure in either gender. In a subgroup of female volunteers, snus caused a decreased stroke index as measured by thoracic electrical bioimpedance. To our knowledge, this is the first study on acute vascular response to Swedish snus assessed using pulse wave velocity, pulse wave analysis and thoracic electrical bioimpedance.

Interestingly, in the current study females showed a deviating response to snus use than males. Snus exposure caused a significantly higher HR and BP elevation as compared to men, even though weekly snus consumption was generally the same in both men and women. Hering *et al*. have previously shown that smoking one cigarette has similar gender differences, namely a higher HR and SBP in women compared to men [25]. Indeed, there are clear gender differences in cardiovascular risk in smokers, with a fifty per cent increased risk of myocardial infarction for female smokers as compared to male smokers [26].

Nicotine stimulates the release of catecholamines from adrenal glands and has a direct sympathomimetic effect on the central nervous system and endothelial function [27, 28]. In general, women have a lower resting autonomic nerve activity and react more to catecholamines than men [29]. This may suggest that gender responses to nicotine could play an underlying key role in the disparate acute hemodynamic responses demonstrated in the current study.

The present study was not primarily designed to investigate gender differences and therefore the results were somewhat surprising. Nevertheless, the observed discrepancy in acute responses to Swedish snus between men and women is highly interesting, as most of the previous cohort studies for the cardiovascular risk of snus use have been solely performed in male subjects [6, 7, 13]. This may be attributed to the fact that the prevalence of snus use is still lower in women as compared to men, as four percent of Swedish women use snus on a daily basis and 5.8 percent of American women report having ever tried snus [30, 31]. However, in the countries where snus is available, there has been a steady increase among female snus users [32]. More extensive data exists for cigarette use and it has been shown that smoking females have a higher relative risk for myocardial infarction than smoking males [26, 33, 34]. It is important to address this gender difference in future studies to properly assess cardiovascular risk for female snus users.

Usage of a snus pouch in the current study gave immediate effects on HR and BP, which is in line with previous findings (Rohani, Agewall 2004). Similar effects have also been demonstrated following smoking of one cigarette [35, 36]. This increase in HR and BP may be attributed to the nicotine content in these tobacco products. Benowitz *et al*. demonstrated that nicotine infusion caused a sudden increase in BP and HR [37]. This theory is further supported by Adamopoulos *et al*. who showed that administration of one tablet of nicotine (2mg) caused similar effects [38]. As mentioned before, nicotine has a direct sympathomimetic effect, which subsequently causes a release of catecholamines [27]. These catecholamines, specifically epinephrine and norepinephrine, are pro-arrhythmogenic and may also have a pro-thrombotic effect [39–42]. Taken together, the high and prolonged nicotine exposure in snus users may explain the higher incidence of fatal stroke and fatal myocardial infarctions in habitual snus users [10, 13]. It may also account for that discontinuation of snus following myocardial infarction reduces mortality by almost fifty per cent [9]. On the other hand, nicotine replacement therapy (NRT) does seem to be safe following myocardial infarction and it has not been shown to increase the risk for cardiovascular disease during a follow-up period of 12 months [43, 44]. One possible explanation may be that Swedish snus generally has a pH in the range of 7.8–8.5 (compared to an average pH of 5.3 in cigarette smoke) [45, 46]. Increasing the pH increases the portion of free base (unprotonated) form of nicotine, which accelerates mucosal nicotine absorption. Therefore snus users tend to have higher and more prolonged nicotine absorption than NRT users due to the high pH in snus [47].

In addition to nicotine, there are several other components found in snus that may cause the demonstrated effect, including polycyclic aromatic hydrocarbons (PAHs) or aldehydes [48, 49]. PAHs are organic chemicals, many of which are classified as carcinogens, which cause damage in the lung tissue when inhaled. However, PAHs can also be ingested or absorbed following dermal and mucosal exposure [50]. It has been suggested that PAHs may affect the redox balance in favor of reactive oxygen species (ROS). ROS cause oxidative damage to biological structures leading to various diseases including cancer, atherosclerosis and cardiovascular disease [51].

In the present study we assessed the acute effects on aortic stiffness and distensibility measured by pulse wave analysis and pulse wave velocity. However, we did not observe any acute changes in AiX75 or PWV. In a similar study, but without placebo-control, AiX75 was shown to increase following exposure to American smokeless tobacco (ASLT) in a group of habitual users [52]. ASLT differs from Swedish snus with higher levels of carcinogens like PAHs and aldehydes due to fire curing during manufacturing [49]. Similar effects with acute increase in arterial stiffness were observed following smoking of one cigarette or administering of one 2mg nicotine tablet [35, 38]. In the present study, we investigated daily snus users following a snus-free period of twelve hours. It may be possible that arterial stiffness is chronically altered in snus users due to differences in nicotine delivery compared to smokers or users of nicotine gum. Snus users tend to have very long exposure times for around 10 hours per day resulting in prolonged high nicotine-levels [47, 53]. Chronic exposure slows down nicotine metabolism as compared to what is seen following occasional exposure [54]. Therefore the prolonged nicotine exposure often seen in snus users may attenuate certain acute effects of nicotine [55].

As demonstrated in the current study, short-term exposure to snus has an immediate impact on cardiac function and systemic vascular resistance measured using thoracic electrical bioimpedance. This non-invasive method is mostly used to quickly assess cardiac function in a critical care setting, yet is also used in outpatient care settings [56, 57]. We observed a sudden decrease in SI and a trend towards increased SVRI following 20 min of snus exposure. This observation was seen at a later time-point than the effects on HR and BP. Sundström *et al*. have previously shown that acute exposure to Swedish snus caused a decrease in diastolic function as assessed by cardioechography [58]. Similar effects on cardiac function were also found following smoking of one cigarette [59]. As discussed before, it is possible that nicotine exerts these acute effects on cardiac function, but other compounds may play a crucial role.

Even though we did not observe any acute effects on arterial stiffness, we found that women had overall higher AiX75, but slightly lower PWV than men. Similar gender differences have been previously reported in healthy volunteers [60–62]. Physiological differences between men and women, like hormonal or constitutional ones have been discussed as possible explanations for this phenomenon [63, 64]. On the other hand, some studies suggest a higher cardiovascular risk for women with elevated arterial index compared to men [65, 66]. Our study participants were habitual snus-users and we observed no differences in snus consumption between males and females. Accordingly, differences in snus consumption cannot explain this finding. The fact that women had overall higher arterial stiffness and a higher acute hemodynamic response to acute snus exposure suggests strongly that the cardiovascular risk for snus using women needs to be further elucidated.

### Limitations

This study was not designed to investigate gender differences. Therefore results have to be interpreted with caution.

Data from two studies was pooled for analysis. Even though we had identical study designs, two separate investigators performed arterial stiffness measurements.

Thoracic electrical bioimpedance measurements were only performed in the six female subjects in study 1. A study involving more study participants including male volunteers would strengthen our thoracic electrical bioimpedance results.

## Conclusions

Brief exposure to Swedish snus causes acute changes in blood pressure and heart rate, however this change is more pronounced in females. The novel finding that snus may have different gender related vascular effects needs to be further investigated, especially as prior health effect studies have mostly focused on males.

## Supporting information

S1 Fig Mean change in systolic and diastolic blood pressure (SBP, DBP). Mean values with standard deviations during 45 minutes of exposure and 30 minutes post exposure to snus or control. P-values are presented for the interaction of time and exposure in multiple measures ANOVA.
(TIFF)

S2 Fig Mean change in arterial stiffness. Mean values for arterial index adjusted for a heart rate at 75 bpm (AiX75) and pulse wave velocity (PWV) with standard deviations during 45 minutes of exposure and 30 minutes post exposure to snus or control, separated for males and females. P-values are presented for the interaction of time and exposure in multiple measures ANOVA.
(TIFF)

S1 Table Baseline characteristics for females in study 1 and 2. SBP = systolic blood pressure, DBP = diastolic blood pressure, HR = heart rate, AiX75 = arterial index for a heart rate at 75bpm, PWV = pulse wave velocity. Expressed as mean values ± SD.
(DOCX)

S2 Table Baseline values prior to exposure. SBP = systolic blood pressure, DBP = diastolic blood pressure, HR = heart rate, AiX75 = arterial index for a heart rate at 75bpm, PWV = pulse wave velocity. Expressed as mean values ± SD.
(DOCX)

S3 Table Mean values ± SD, (mean changes ± SD from baseline) separated by gender. SBP = systolic blood pressure, DBP = diastolic blood pressure, HR = heart rate, AiX75 = arterial index for a heart rate at 75bpm, PWV = pulse wave velocity. Early exposure = 0–20 minutes of exposure, late exposure = 20–45 minutes of exposure, post exposure = 0–30 minutes post exposure. P-values for mean change from baseline: *<0.001, †<0.01, ††<0.05.
(DOCX)

S1 Dataset SPSS dataset: Snus study 1. (SAV)

S2 Dataset SPSS dataset: Pooled analysis. (SAV)
