# Supplementary figures and images for: Brief exposure to Swedish snus causes divergent vascular responses in healthy male and female volunteers
Source: PLoS One. 2018 Apr 18;13(4):e0195493. doi: 10.1371/journal.pone.0195493 (PMC5905986; doi:10.1371/journal.pone.0195493)

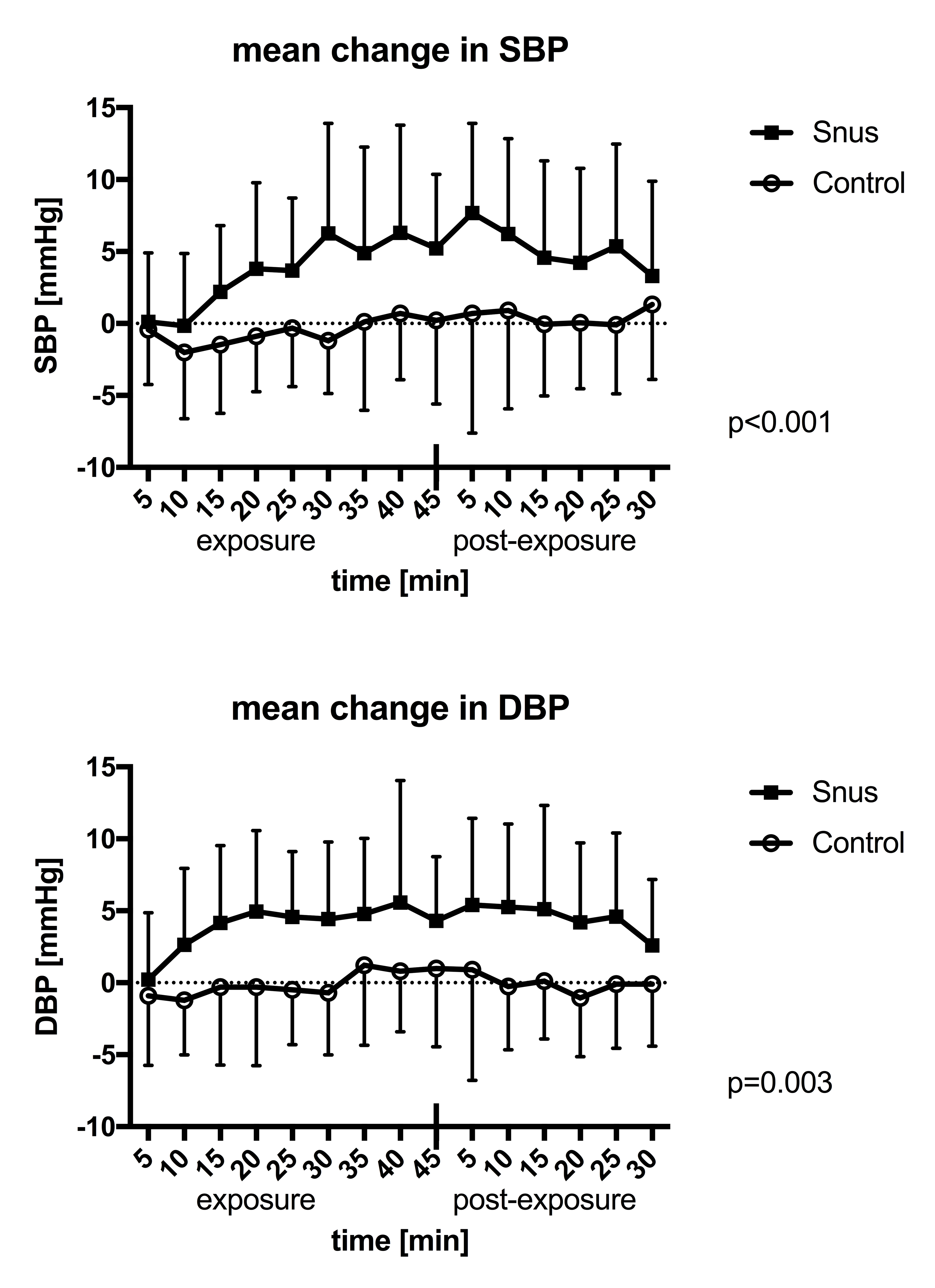

Supplement: S1 Fig — Mean values with standard deviations during 45 minutes of exposure and 30 minutes post exposure to snus or control. P-values are presented for the interaction of time and exposure in multiple measures ANOVA. (TIFF) [file pone.0195493.s001.tiff]

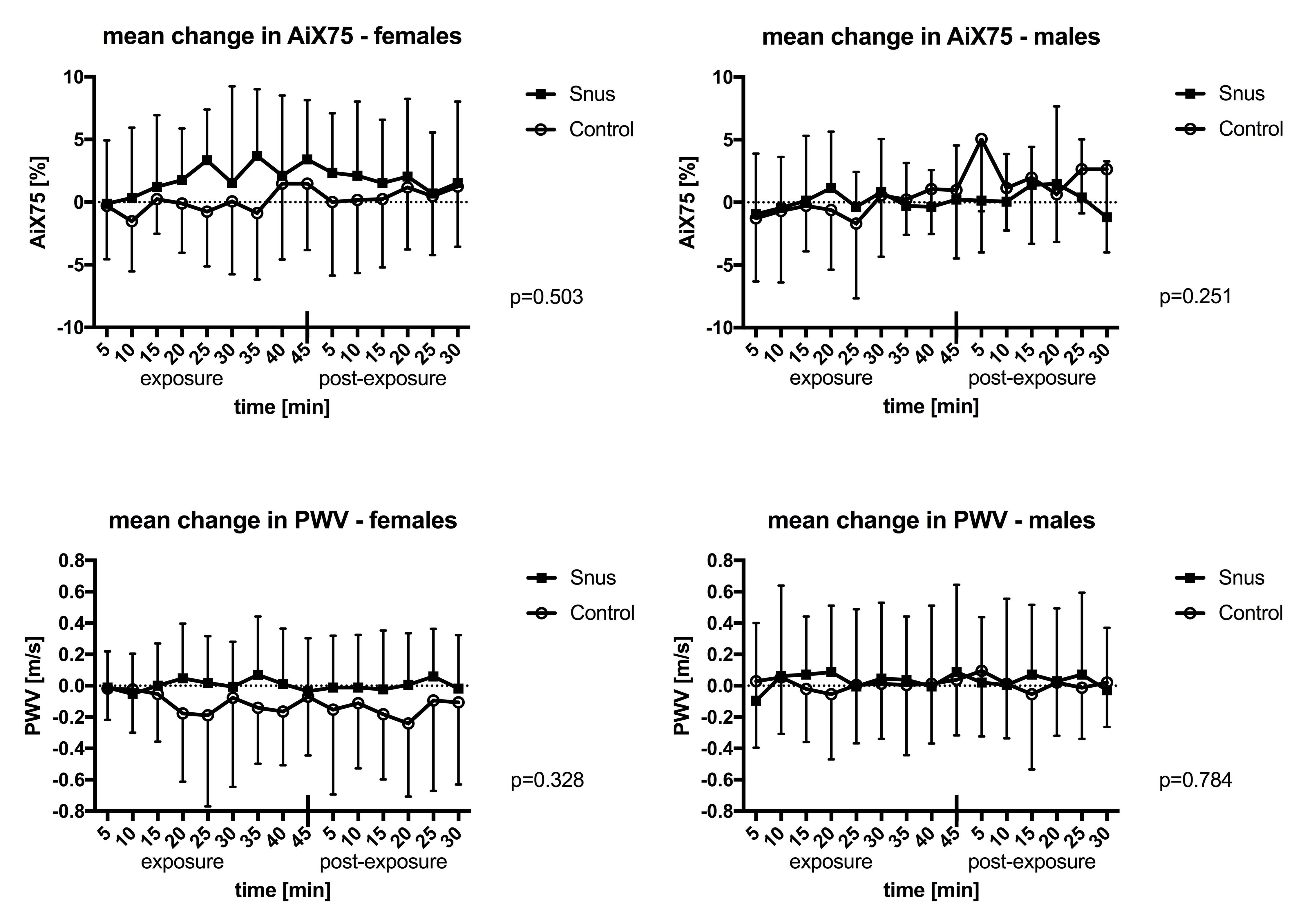

Supplement: S2 Fig — Mean values for arterial index adjusted for a heart rate at 75 bpm (AiX75) and pulse wave velocity (PWV) with standard deviations during 45 minutes of exposure and 30 minutes post exposure to snus or control, separated for males and females. P-values are presented for the interaction of time and exposure in multiple measures ANOVA. (TIFF) [file pone.0195493.s002.tiff]
