# Supplementary material for: Brief exposure to Swedish snus causes divergent vascular responses in healthy male and female volunteers
Source: PLoS One. 2018 Apr 18;13(4):e0195493. doi: 10.1371/journal.pone.0195493 (PMC5905986; doi:10.1371/journal.pone.0195493)
Supplement: S1 Table — SBP = systolic blood pressure, DBP = diastolic blood pressure, HR = heart rate, AiX75 = arterial index for a heart rate at 75bpm, PWV = pulse wave velocity. Expressed as mean values ± SD. (DOCX) [file pone.0195493.s003.docx]

|  | Study 1  (females: n=6) | Study 2  (females: n=11) | p-values |
| --- | --- | --- | --- |
| Age [years] | 27.3 ± 7.8 | 29.4 ± 5.6 | n.s. |
| Snus use [years] | 12.2 ± 6.1 | 11.9 ± 5.6 | n.s. |
| Snus [cans/week] | 4.6 ± 2.8 | 5.4 ± 1.3 | n.s. |
| SBP [mmHg] | 110.6 ± 8.7 | 107.8 ± 7.7 | n.s. |
| DBP [mmHg] | 68 ± 7.3 | 67 ± 7.1 | n.s. |
| HR [bpm] | 57.1 ± 11.1 | 56.7 ± 9.3 | n.s. |
| AIx75 [%] | 1 ± 6.8 | -0.2 ± 11.6 | n.s. |
| PWV [m/s] | 5.3 ± 0.8 | 5.6 ± 0.7 | n.s. |
