# Supplementary material for: Brief exposure to Swedish snus causes divergent vascular responses in healthy male and female volunteers
Source: PLoS One. 2018 Apr 18;13(4):e0195493. doi: 10.1371/journal.pone.0195493 (PMC5905986; doi:10.1371/journal.pone.0195493)
Supplement: S2 Table — SBP = systolic blood pressure, DBP = diastolic blood pressure, HR = heart rate, AiX75 = arterial index for a heart rate at 75bpm, PWV = pulse wave velocity. Expressed as mean values ± SD. (DOCX) [file pone.0195493.s004.docx]

|  | control | snus |
| --- | --- | --- |
| SBP [mmHg] | 113.4 ± 10.2 | 113.7 ± 10.6 |
| DBP [mmHg] | 69.1 ± 7.7 | 68.7 ± 7.9 |
| HR [bpm] | 54.7 ± 10.6 | 56.4 ± 11.0 |
| AIx75 [%] | -4.5 ± 10.3 | -4.3 ± 10.3 |
| PWV [m/s] | 5.8 ± .8 | 5.8 ± .7 |
