# Supplementary material for: Brief exposure to Swedish snus causes divergent vascular responses in healthy male and female volunteers
Source: PLoS One. 2018 Apr 18;13(4):e0195493. doi: 10.1371/journal.pone.0195493 (PMC5905986; doi:10.1371/journal.pone.0195493)
Supplement: S3 Table — SBP = systolic blood pressure, DBP = diastolic blood pressure, HR = heart rate, AiX75 = arterial index for a heart rate at 75bpm, PWV = pulse wave velocity. Early exposure = 0–20 minutes of exposure, late exposure = 20–45 minutes of exposure, post exposure = 0–30 minutes post exposure. P-values for mean change from baseline: *<0.001, †<0.01, ††<0.05. (DOCX) [file pone.0195493.s005.docx]

|  |  | snus | | control | |
| --- | --- | --- | --- | --- | --- |
|  |  | females | males | females | males |
| SBP | baseline | 108±8.5 | 119.8±8.7 | 108.6±8 | 119.4±9.8 |
| [mmHg] | early exposure | 111.2±9 (3.2±3.2)† | 122±8.1 (2.2±4.2)† | 107.9±7.8 (-0.7±2) | 118.8±11.2 (-0.6±3.5) |
|  | late exposure | 115.6±10.1 (7.6±4.8)* | 125.7±8.7 (5.9±4.9)†† | 109±7.9 (0.3±3.9) | 120.1±10.6 (0.6±4.6) |
|  | post exposure | 115±9 (7±4.7)* | 124.5±10 (4.7±6)†† | 109.2±6.8 (0.6±3.3) | 120.5±9 (1.1±5.5) |
| DBP | baseline | 66.8±6.9 | 70.9±7.9 | 67.1±6.9 | 70.2±8.3 |
| [mmHg] | early exposure | 71.3±7.3 (4.5±3.5)* | 73±8.3 (2.1±3.5)† | 67.5±6.5 (0.5±3) | 69.7±8.1 (-0.5±2.5) |
|  | late exposure | 72.5±7.2 (5.7±4.3)* | 75.1±8.2 (4.2±4.4)† | 68.1±6.7 (1±2.8) | 72±8.4 (1.9±4.3) |
|  | post exposure | 72.1±6.9 (5.3±4.5)* | 74.8±9.2 (3.9±4.3) | 67.3±6.3 (0.3±2.2) | 71.3±7.8 (1.1±2.9) |
| HR | baseline | 56.5±9.8 | 56.8±12.4 | 57.6±9.8 | 51.3±10.4 |
| [bpm] | early exposure | 65.9±11.4 (9.4±4.7)* | 59.4±12.5 (2.6±6.6) | 57.7±9.8 (0.1±3.9) | 51.6±9.1 (0.3±1.5) |
|  | late exposure | 68.4±11 (11.9±4.5)* | 61±10.5 (4.2±6.4)†† | 58.1±9.8 (0.5±2.8) | 52.8±9.4 (1.5±2.8) |
|  | post exposure | 66.7±11.2 (10.1±4.6)* | 59.9±10.3 (3.1±8) | 57.8±9.8 (0.2±4.2) | 51.6±9.2 (0.3±3.3) |
| AiX75 | baseline | -0.1±10.5 | -10.3±6.6 | 0.5±9.9 | -11.6±5.8 |
| [%] | early exposure | 1.2±9.9 (1.3±3.8) | -10.4±6.6 (-0.1±2.7) | 0±9.7 (-0.5±2.6) | -12.6±6.4 (-0.9±3.9) |
|  | late exposure | 2.5±11 (2.6±4.6)†† | -10.2±6.9 (0.1±2.5) | 0.9±8.9 (0.4±3.8) | -10.1±5.3 (1.6±2.3)†† |
|  | post exposure | 1.6±10.6 (1.7±4.6) | -9.9±7.5 (0.4±3.6) | 1.1±8.9 (0.6±4) | -9.5 (2.3±3.3)†† |
| PWV | baseline | 5.4±0.6 | 6.3±0.5 | 5.6±0.9 | 6.3±0.5 |
| [m/s] | early exposure | 5.4±0.5 (0±0.2) | 6.3±0.5 (0±0.4) | 5.4±0.8 (-0.1±0.3) | 6.3±0.4 (0±0.3) |
|  | late exposure | 5.4±0.5 (0±0.3) | 6.3±0.4 (0±0.4) | 5.4±0.8 (-0.1±0.4) | 6.3±0.4 (0±0.3) |
|  | post exposure | 5.4±0.6 (0±0.3) | 6.3±0.4 (0±0.4) | 5.4±0.7 (-0.2±0.5) | 6.3±0.4 (0±0.3) |
